# Editorial

**Published:** 1891-02

**Authors:** 


### Editorial.

### CREMATION.

Science has led us out of wrong beliefs and opinions, overturned the superstitions of centuries and reflected the light of truth upon the mysterious phenomena of mind and matter.

It is a school master to lead us upward and onward into all the recesses of knowledge, whether of atoms or of words; of man or of Jehova. The indestructibility of matter, fairly proven, suggests the indestructibility of the soul. The acknowledgment, that man was created by the Deity, whether out of the dust of the earth or by evolution of protoplasm, is a virtual admission, that, though he may be changed, he cannot die eternally. Yet man does die corporeally; or rather the organs of the body, especially the brain, not being able to respond to action of the vis vitae, dissolution takes place. The remains soon begin to decay and now comes the *question* of *disposal.* The mortal dread of burying our friends alive causes us to entertain misgivings, if we inter too early, and exposes us to great danger, if interment be delayed. To be buried alive is a horrible thought; but to poison a whole community by exhalations from an exanimate body, is also terrible. Science comes to our aid and the electric battery tells the tale of life or death, and all doubt is removed in the case.

Science again comes to us, laden with facts as to the best and safest disposition of the dead. Cremation is the most scientific and philanthropic. The dead cannot be hurt. The living should be protected. Every grave-yard is a center of infection. The germs of disease continue to multiply while the body is decaying and putrefying in the ground, and find their way into the water which we drink, and the atmosphere which we breathe. Thus the most fearful endemics arise and scourge the people. This is, in our opinion, the cause of the malignancy of fevers, pneumonias, dysenteries, etc., in our cities, especially those whose system of sewerage is bad. Of course, the latter is on the same line and in itself a sufficient cause. Cemeteries are often so situated, as to endanger the water supply, and there are none whose atmosphere is not more or less contaminated. Fire is the great distroyer of germ life. It is the great purifier.

It is said that finally the "elements shall melt with fervent heat, the earth also and the works that are therein shall be burned up. "Nevertheless, we, according to His promise, look for new heavens and a new earth wherein dwelleth righteousness." I can say that we also look forward to the time when the enemies to human life shall, for the most part, be destroyed through the improved methods of hygiene. The light of civilization shines more brightly every day. It exposes the fallacies of the ages and places mankind on a higher plane of existence.

It will soon destroy the prejudice against cremation. All liberal-minded, intelligent and thinking people will agree, that this is thus far, the safest and least revolting of all other methods. Christ said that the "whited sepulchers" "indeed appear beautiful outward, but are, within, full of dead men's bones and of *all uncleanness.*"

It is time the people should be educated out of the deadly customs of the past and be led into other and more inviting fields of thought and action on this momentous question.

A. W. G.

### PHILADELPHIA POLYCLINIC.

We have received the report of the Trustees of the Philadelphia Polyclinic and College for Graduates in Medicine.

It is with pleasure that we note the success of this school, both in its hospital work and in its teaching department. We wish long life and prosperity to the Philadelphia Polyclinic.

### THE AMERICAN ACADEMY OF MEDICINE.

This society is one of the best in the country, but its objects and purposes are, we fear, misunderstood by the majority of the profession, who seem to think much of a class of physicians who desire to set themselves above their fellow-practitioners. This is wrong, and any one can see for themselves if they will read the Constitution and By-Laws which they can obtain by writing Dr. R. J. Dunglinson, 814 North 16 street, Philadelphia, Penn.

This Society thus briefly sets forth its objects in the Constitution:

1. To bring those who are Alumni of Classical, Scientific and Medical Schools into closer relations with each other.

2. To encourage young men to pursue regular courses of study in Classical and Scientific Institutions before entering upon the study of Medicine.

3. To extend the bounds of Medical science, to elevate the profession, to relieve human suffering, and to prevent disease.

### THE ATMOSPHERIC TRACTOR..

This instrument for use in obstetrics promises to fill a long felt want. We have not had the opportunity to try it yet, but it seems as if it should prove of the greatest service in this branch of medicine. It is the invention of Dr. Peter McCahey, No. 219 North 22d street, Philadelphia.

### A REQUEST TO OUR READERS.

Throughout the coming year, in each issue of the Record, we desire to print letters from our subscribers, giving their treatment of various diseases. It is desirable that these letters be as short and concise as possible, so that there can be a chance for all to be heard. We would suggest for our next series "Pneumonia", this to be followed by a series on "Diphtheria." We hope that in this new departure all will join hands and make it as interesting as possible.

### Dr. E. V. JOYE.

It is with the greatest sorrow that we report the death of Dr. Edward V. Joye, which occurred on Wednesday morning, Jan. 21st, from pneumonia. The Doctor was about forty years old and was born in Charleston, S. C.

He was a man of fine attainments and justly popular with those who knew him. He was a member of The Atlanta Society of Medicine, The Fulton County Confederate Veteran Association, and a thirty-second degree mason.

---

### TO OUR READERS.

We wish to call your special attention to the following *New Advertisements* we received during the months of January and February We hope you will read them, as we know you can find whatever you need from them.

I. O. Woodruff & Co.
Fairchild's Bros. & Foster.
E. Fougera & Co.
C. N. Crittenton & Co.
The Tyndale Eucalyptus Co.
T. C. Morgan & Co.
Dr. Strong's Sanitarium.
Dr. G. W. Hartshorne.
D. F. Sargent & Son.
Pneumatic Tampon Co.
E. C. Allen.
Dr. H. A. Mumaw.
M. P. Delgardo.
True & Co.
H. Hallet & Co.
Moore-McGregor.
W. G. Farrar.
Stinson & Co.
Munn & Co.
Roberts & Allison.
Dr. Hugh Hagan.
Joseph Thompson.
J. S. Ogilvie.
Antikamnia Chemical Co.

